# Mutations at the *SLC20A2* gene and brain resilience in families with idiopathic basal ganglia calcification (“Fahr's disease”)

**DOI:** 10.3389/fnhum.2013.00420

**Published:** 2013-08-05

**Authors:** Danyllo F. de Oliveira, Roberta R. de Lemos, João R. M. de Oliveira

**Affiliations:** ^1^Keizo Asami Laboratory, Federal University of PernambucoRecife, Brazil; ^2^Neuropsychiatry Department, Federal University of PernambucoRecife, Brazil

Wang et al. (2012) recently identified seven novel mutations at the *SLC20A2* gene in families from China, Spain and Brazil, suggesting that Familial Idiopathic Basal Ganglia Calcification (IBGC) might be a phosphate imbalance disorder. Expressing wild-type human *SLC20A2* and the mutated variants, using transport assays in *Xenopus* oocytes, resulted in significant impaired Pi transport. On the other hand, mutants did not have an obvious effect on the Pi transport activity when co-expressed with the wild-type PiT2 protein, encoded by *SLC20A2*. This suggests that such mutations have an effect through haploinsufficiency (Wang et al., 2012).

Additional analysis showed that this gene is responsible for ~50% of the familial cases (Hsu et al., 2013; Lemos et al., 2013). More recently, two French families with IBGC were reported with mutations at the platelet derived growth factor receptor B (PDGFRB) gene, opening now a new venue for the comprehension of this phenotype as being caused by different molecular failures in mechanisms of vascular homeostasis (Nicolas et al., 2013).

Familial or sporadic IBGC, often called by the misnomer “Fahr's Disease,” is a neuropsychiatric disorder with variable clinical outcome, including parkinsonism, psychosis, dementia and headaches. The diagnosis criteria include bilateral calcifications, often documented with computerized tomography, in the absence of hormonal and metabolic imbalances. Such subjects are often misdiagnosed as patients with schizophrenia, Parkinson's disease, bipolar Disorder, and Alzheimer's disease (Manyam, 2005; Lemos et al., 2011).

Calcifications might also be detected in cerebellum, thalamus and cortex. The mode of inheritance of IBGC is mainly reported in literature as autosomal dominant, sometimes displaying anticipation, a phenomenon widely described in diseases with similar pattern of inheritance and first reported in familial IBGC by Geschwind et al. (1999).

Families affected with IBGC also show a variable clinical manifestation and a lack of full penetrance. Recent functional studies aiming to access the expression profiles of *SLC20A2* at the kidney, have reported that the expression of this gene is tightly linked to inorganic phosphate (Pi) concentration into the organism. Moreover, together with *SLC20A1*, these genes act at the Pi uptake under different conditions of pH and dietary Pi (Villa-Bellosta et al., 2009).

Calcification is an unspecific biochemical process occurring normally even under physiological conditions, through phosphate deposition and hydroxyapatite crystals formation, similar to the mineral fraction of the bones. However, disturbances in this process, which are often related to phosphate transport alterations at epithelial cells of blood vessels, lead to calcification around these tissues, jeopardizing the vessels' elasticity and leading to arteriosclerosis as well (Villa-Bellosta and Sorribas, 2011).

Curiously, Wang et al. (2012) and other authors also reported subjects with calcifications but no symptoms. Brain resilience for such lesions is still the most compelling challenge for the complete understanding of the pathological mechanisms of IBGC and others autosomal dominant neuropsychiatric conditions. Argylean et al. (2009) used neuroimaging techniques to assess the cerebellumthalamocortical connectivity (CbTC) in individuals with dystonia and compared these data with that was yielded by the analysis of this same circuit in control individuals and asymptomatic carriers of mutations for dystonia. Their analysis showed reduced integrity of cerebellothalamocortical fiber tracts, in both clinically manifesting and nonmanifesting mutation carriers. In these subjects, reductions in cerebellothalamic connectivity were associated with increased motor activation responses, consistent with loss of inhibition at the cortical level. Nonmanifesting mutation carriers displayed an additional area of fiber tract disruption, distally situated along the thalamocortical segment, in tandem with a proximal cerebellar outflow abnormality. In individual gene carriers, clinical penetrance was determined by the difference in connectivity measured at these two sites.

Other approaches to investigate the lack of full penetrance in genetic traits have been based on functional studies using the one-thousand-cells nematode *Caenorhabditis elegans*. These analyses state that this phenomenon may occur due to mitigating mechanisms involving “genetic interaction partners,” that is, genes involved in common molecular networks (Burga et al., 2011).

On this perspective, two molecular mechanisms have been described: the first one involves a high expression of homologous genes, which because of its similar coding sequence, still bears related functions, enabling them to play the role which should have been done by the damaged gene. The other mechanism involves Heat Shock Proteins, which, by folding proteins into its biologically active conformation, ends up attenuating the effects of mutations along the peptidic sequence (Burga et al., 2011).

The haploinsufficiency effect described by Wang et al. (2012) when expressing SLC20A2 mutations in *Xenopus* oocites lead us to speculate that in asymptomatic carriers of those deleterious mutations must have an altered pattern of expression of paralog genes. This assumption would be in accordance with the first mechanism described by Burga and colleagues (2011). Functional studies in model organisms may be able to test this hypothesis.

Previous studies with clinical heterogeneity of familial IBGC shows that ~40% of the patients carrying basal ganglia calcification do not show symptoms. We reported previously that symptoms were present in only 28 patients with IBGC, from a total of 47 subjects with calcifications detected during a linkage analysis of 6 families (Oliveira et al., 2003). This suggests that the brain might have mechanisms to handle calcifications and the first symptoms outcome (resilience), however, we cannot exclude the possibility that we are still missing the precocious detection due to less sensitive clinical findings. On the other hand, it is well established that studies measuring the total volume of calcification shows far more deposits in symptomatic individuals compared to asymptomatic subjects (Manyam, 2005).

We suggest considering two levels of penetrance in IBGC: one for the calcification formations and another for the clinical manifestation but only a proper follow-up of asymptomatic subjects with calcifications will detect the exact timing of the appearance the first symptoms and will define the limits of brain resilience to calcifications. An animal model will most likely provide this answer.

The concept of brain resilience is intrinsically associated to IBGC pathophysiology, since this is the most probable mechanism underlying the absence of clinical manifestations pathogenic SCL20A2 mutation carriers. This lack of concordance between the genetic and phenotypic statuses, gives rise to the probabilistic concept of penetrance in human genetics, that is, the likelihood of a given damaging genotype turns out to develop a clinical entity in its carriers.

As for IBGC, the concept of incomplete penetrance should be further elaborated, in order to better categorize phenotipically the individuals present in a given heredogram. On this perspective, we propose the term “radiological penetrance.”

Imaging genetics is a promising strategy to integrate genotypic and phenotypic data and more studies—especially those focusing brain imaging in asymptomatic IBGC carriers—are necessary to properly test such hypotheses.
